# *Helicobacter pylori* Infection and Gastric Dysbiosis: Can Probiotics Administration Be Useful to Treat This Condition?

**DOI:** 10.1155/2018/6237239

**Published:** 2018-09-10

**Authors:** Giovanni Bruno, Giulia Rocco, Piera Zaccari, Barbara Porowska, Maria Teresa Mascellino, Carola Severi

**Affiliations:** ^1^Department of Internal Medicine and Medical Specialties, Gastroenterology Unit, Sapienza University, Rome, Italy; ^2^Department of Cardio-Thoracic, Vascular Surgery and Transplants, Sapienza University, Rome, Italy; ^3^Department of Public Health and Infectious Diseases, Sapienza University, Rome, Italy

## Abstract

*Helicobacter pylori* (*Hp*) is responsible for one of the most common infections in the world. The prevalence exceeds 50% of the population in developing countries, and approximately one-third of the adults are colonized in North Europe and North America. It is considered a major pathogenic agent of chronic gastritis, peptic ulcer, atrophic gastritis, gastric cancer, and mucosa-associated lymphoid tissue lymphoma (MALT). *Hp* colonization modifies the composition of gastric microbiota that could drive the development of gastric disorders. Currently, an emerging problem in *Hp* treatment is represented by the increasing rate of antimicrobial therapy resistance. In this context, the search for adjuvant agents can be very useful to overcome this issue and probiotics administration can represent a valid option. The aim of this review is to describe the gastric microbiota changes during *Hp* colonization, the mechanisms of action, and a possible role of probiotics in the treatment of this infection.

## 1. Introduction

*Helicobacter pylori* (*Hp*) is a Gram-negative, spiral-shaped, flagellated bacterium belonging to Proteobacteria phylum with a strong capacity of surviving in the harsh acid milieu of the stomach. *Hp* is responsible for one of the most widespread infections in the world [1, 2], and the number of infected subjects is still very high worldwide. The prevalence exceeds 50% of the population in some areas of the world, like South and East Europe, South America, and Asia. Low socioeconomic status, bad level of education, and poor hygienic conditions are the major related risk factors. Notably, about one-third of the adults are colonized in North Europe and North America [3].

Several recent evidence have highlighted that *Hp* can modify the composition of gastric microbiota and the resulting changes can play a role in the development of *Hp*-related diseases. Still, the interaction between the host, microbiota, and *Hp* in the pathogenesis of these conditions has to be fully elucidated [4].

*Hp* colonization can cause chronic gastritis, peptic ulcer, atrophic gastritis, gastric adenocarcinoma, and mucosa-associated lymphoid tissue lymphoma (MALT) [5, 6]. *Hp* gastritis is considered an infectious disease regardless of symptoms and disease stage, and eradication therapy is strongly recommended [7]. However, a rapid emergence of antibiotic-resistant bacteria is becoming one of the world's most critical public health problems, and thus, the choice of therapeutic options for the treatment of *Hp* infection faces this dilemma [8].

In this context, the use of probiotics, defined as “live microorganisms which when administrated in adequate amounts confer a health benefit on the host” [9] can be helpful for their antibacterial activity against *Hp* and for the interaction with the complex ecosystem of the host [10].

The beneficial properties of probiotics on the host microbiological environment can be associated with their potential effects on digestive microflora and gut immune system that include their ability to compete with gut pathogens, to increase IgA secretion, to modulate cytokine mRNA expression and secretion, to stimulate mucin, bacteriocin, and lactic acid production, and to modulate microbiota growth [11–13].

The aim of this review is to provide an overview of the changes in gastric microbiota composition during *Hp* infection and then to assess the potential role of probiotics in *Hp*-induced dysbiosis and eradication. A critical appraisal of the clinical research evidence on the data regarding *Hp* and gastric microbiota composition and on the probiotics effectiveness to treat *Hp* infection and to prevent antimicrobial therapy side effects was made. The search was limited to full manuscripts in English language.

## 2. *Helicobacter pylori* and Gastric Microbiota Composition

The stomach has always been considered a sterile organ. It is not surprising that for long, it was believed that low pH of the gastric lumen and peristalsis contributed to create an adverse environment for bacterial survival and stable microbial colonization of this organ. However, in 1983, the discovery of *Hp* by Marshall and Warren [14] gave the input to a period of progressive discoveries in the field of gastric infection and ensured a breakthrough in understanding gastric microecological environment. The improvement in microbial detection techniques has been crucial. The initial analysis was performed using culture-based methods, which harbor various limitations; in particular, they regard a large amount of bacteria that are still considered “unculturable” due to growth resistance in conventional culture media, need for particular environment conditions, low bacterial growth rate, and interaction with other bacteria or their secreted substrates [15]. Taken together or individually, these factors can determine an incomplete and limited representation of the complex gastric bacterial community improperly showing a similar gastric microbiota composition in *Hp* patients compared with healthy subjects [16, 17]. Conversely, the most recent molecular techniques which allow an in-depth study of the gastric microbiota have highlighted a significant difference in microbiota composition between *Hp*-positive and healthy subjects. The technique consists in sequencing the ribosomal gene 16S rRNA that contains 9 variable regions, which are present in all bacteria and are similar in the microorganisms of the same phyla. Technically, this type of analysis allows the recognition of different bacterial species through the analysis of the genoma, which makes it difficult to evaluate the vitality of microorganisms. To avoid this drawback, it is preferable to use bacterial RNA instead of DNA [18].

Currently, we know that, in healthy subjects, gastric microbiota is composed mainly of Firmicutes, Bacteroidetes, Proteobacteria, and Actinobacteria at phyla level. The most present genera are *Streptococcus*, followed by *Veillonella*, *Prevotella*, *Fusobacterium*, and *Rothia* [17, 19]. The analysis performed on gastric juice and biopsies has suggested that the density of gastric microbiota is lower than in other parts of the gastrointestinal (GI) tract, counting about 10^1^–10^3^ CFU/ml [20].

The presence of bacteria in the stomach is possible due to the progressive shift of pH from the gastric lumen (pH 1-2) to the mucosal surface (pH 6-7) coated with mucus which is actively secreted by gastric glands. This pH gradient permits the development of different environments that allow the growth of microorganisms, being the mucosal surface the more hospitable gastric area [21]. *Hp* colonization of gastric mucosa alters this gastric habitat by deconstructing the mucous layer and by alkalinization of gastric juice [22].

In *Hp*-positive subjects, molecular analysis showed an overall decrease in bacterial diversity with the absolute prevalence at phyla level of Proteobacteria, followed by Firmicutes, Bacteroidetes, and Actinobacteria. In the genus subanalysis, excluding *Hp* which turns out to be the most represented species, *Streptococcus* are commonly the second detected bacteria [23, 24]. An interesting study by Andersson et al., evaluating samples from both, *Hp*-positive and healthy subjects, showed that the presence of *Hp* causes a reduction of microbial diversity. Only 33 phylotypes were found in *Hp* subjects compared to a rich and diversified assortment of gastric microorganism of about 262 phylotypes in healthy people, highlighting this difference [25].

In order to establish whether gastric microbiota alterations found in concomitant with *Hp* infection were induced by hypochlorhydria, Parsons et al. analyzed gastric microbiota composition in various hypochlorhydric conditions and in healthy subjects. As previously described, also in this study the stomach of healthy subjects contains the widest bacterial diversity compared with other groups. Interestingly, PPI group shows a microbiota characterization which is similar in a healthy stomach, despite the hypochlorhydric status due to the drug use. A significant difference in microbial diversity was found comparing healthy subjects with *Hp*-related conditions and an interesting data emerge from the comparison between *Hp* gastritis and *Hp*-related atrophic gastritis. In fact, no significant differences were found between these two conditions, suggesting that, in the *Hp* subgroup patients, the role of bacteria may be superior in inducing changes in the composition of gastric microbiota compared with hypochlorhydria [24].

The changes in gastric microbiota due to *Hp* infection can be related to intrinsic properties of bacteria. In fact, *Hp* is provided with oxidase, catalase, and urease activities. The urease is a metalloenzyme able to convert urea into ammonia and bicarbonate that is responsible for the local increase in gastric pH and the safe passage into the gastric lumen. *Hp* flagella enable the pathogen to pass through the mucus layer driven by the pH gradient, thus permitting gastric mucosal invasion. Once established, *Hp* exposes adhesins (HopQ, HopP, and HopS) that permit a receptor-specific close adhesion to gastric epithelial cells and consequently the expression of *CagA* and *VacA* virulence factors which mediate the cytotoxic activity [26–28]. Mucosal damage attracts polymorphonuclear and other immune cells that likely contribute to gastric damage by producing cytokines and other proinflammatory substances [29]. Probably, these factors creating a hostile environment make difficult the survival of the other bacteria, allowing the establishment of gastric dysbiosis.

## 3. *Helicobacter pylori* Eradication and Probiotics Administration

According to the Maastricht V Consensus Report, the standard triple therapy, proton pump inhibitor (PPI) + amoxicillin (AMX) + clarithromycin (CLR), is considered the first-line empirical treatment in low CLR resistance areas. When the regional resistance to CLR is high, considering 15% of population as the verge, nonbismuth quadruple therapy with PPI + AMC + metronidazole (MTZ) + CLR is recommended. In all these cases, a bismuth quadruple therapy can be used as an alternative and it is recommended as first-line treatment in areas with high dual CLR-MTZ resistance. The benefit of *Hp* eradication has been demonstrated in conditions like peptic ulcer disease, MALT lymphoma, iron deficiency anemia, idiopathic thrombocytopenic purpura, and vitamin B12 deficiency [30]. Antibiotic resistance is leading to increasing estimates of treatment failures, as demonstrated by the raising rate of CLR-resistant strains that has reached 40–50% in some areas of the world [8].

Based on the literature data (Table 1), it emerges that some probiotics associated with antibiotic therapy can improve *Hp* eradication rate and moreover, can reduce deleterious side effects due to antimicrobial therapy such as nausea, vomiting, diarrhea, abdominal pain, bloating, and taste disturbance that occasionally are responsible for the withdrawal of the treatment [31, 32].

The role of *Lactobacillus* strain administration in the treatment of *Hp* infection and in preventing antimicrobial therapy side effects has been well documented. The supplementation of these probiotics can directly reduce *Hp* growth rate and *Hp* colonization. In a prospective, randomized, controlled trial by Ojetti et al., adding a probiotic (*L. reuteri* 1 × 10^8^ CFU tid for 14 days) to levofloxacin-based second-line therapy showed an additional 20% eradication rate compared with antibiotic treatment alone, followed by a consistent reduction of symptoms such as diarrhea and nausea [33]. Armuzzi et al. enrolled 120 asymptomatic *Hp*-positive subjects which were randomly assigned to two treatment groups: one receiving anti-*Hp* triple therapy (PPI 40 mg bid, CLR 500 mg bid, and tinidazole 500 mg bid) and the other receiving the same antibiotic therapy associated with *Lactobacillus* GG-containing probiotic (6 × 10^9^ of viable bacteria) bid for 14 days. The analysis documented an improvement in gastrointestinal symptoms in the probiotic supplementation group with a significant reduction in taste disturbances (*p*=0.007), bloating (*p*=0.01), and diarrhea (*p*=0.02). On the other hand, no significant differences in eradication rates were reported (group 1 vs. group 2; PP: 80.7% vs. 80%, *p*=0.9) [34]. Excellent results can be obtained also by providing drinkable food supplements containing *Lactobacilli* or its culture supernatant [35–38].

The beneficial effects of *Bifidobacterium* administration were analyzed by Chitapanarux et al. in a double-blind, placebo-controlled trial using *B. longum* in addition to the standard triple therapy. The results showed significant beneficial effect on *Hp* eradication rate (PP: 28/30, 93.33% vs. PP: 22/30, 73.33%, *p*=0.04) and on diarrhea frequency reduction (25% vs. 3.23%, *p*=0.027) with no significant outcomes on nausea (18.75 vs. 12.90%), taste disturbance (15.63% vs. 12.90%), and epigastric pain (6.25 vs. 3.23%) [39]. However, in a previous study by Yaşar et al. that included 76 histopathologically proven *Hp*-positive patients, the addition of *Bifidobacterium DN-173-*containing yogurt to the standard triple therapy for 14 days resulted in the eradication rate of 66% compared to 53% with antibiotic therapy alone. Anyhow, the increase of *Hp* eradication rate was not statistically significant [40].

*Saccharomyces boulardii*, a yeast probiotic, was particularly effective in reducing side effects of the eradication therapy. Song et al. reported that adding this probiotic to CLR and AMX-based triple therapy for 4 weeks reached 85.4% eradication rate compared to 80% in the absence of probiotic. Gastrointestinal side effects, in particular diarrhea, were more common in the latter group (*p* < 0.05) [41]. A meta-analysis by Szajewska et al. including eleven RCTs (2200 participants, among them 330 children) showed that *S. boulardii* significantly increased *Hp* eradication rate but below the aimed level. However, this probiotic significantly reduced side effects, in particular diarrhea and nausea [42].

Based on the properties of probiotics, it is plausible that a mixture of strains can improve *Hp* eradication rate. In a trial of Du et al., a multistrain probiotic containing *Lactobacillus acidophilus*, *Streptococcus faecalis*, and *Bacillus subtilis* was administered to the patients for two weeks before antibiotic treatment or in another group for two weeks after the eradication therapy. Both schedules were more effective in eradicating *Hp* infection than the triple therapy alone (81.6% and 82.4% vs. 61.5%), but there was no statistical significance regarding the incidence of side effects [43]. A meta-analysis by Wang et al. which included ten trials (1469 subjects) showed that probiotic addition using compounds containing *Lactobacillus* and *Bifidobacterium* strains significantly improves both *Hp* eradication rates and reduces the incidence of antimicrobial therapy side effects. Moreover, the advantage of probiotics supplementation was demonstrated independently of the type of eradication therapy used in the trial, despite the bismuth-containing therapy which could represent a potential failure due to the inhibitory activity of bismuth against the probiotics [44]. Furthermore, a successive meta-analysis by Mcfarland et al. confirmed that multistrain probiotics can be helpful as an adjunct therapy for *Hp* eradication and in preventing eradication side effects, but they concluded that not all mixtures were equally effective [45]. Finally, Lau et al. confirmed the utility of *Lactobacillus*, *Bifidobacterium*, *Saccharomyces*, and probiotics mixtures in the treatment of *Hp* infection in both adults and children, in Asian and non-Asian population [46].

At present, the use of probiotics in children to treat *Hp* infection is not clear. In the meta-analysis previously mentioned by Wang et al., the children subgroup analysis showed no probiotic efficacy on eradication rates and reduction of undesirable side effects [44]. Pacifico et al. pointed out the controversial efficacy of probiotics use in these patients. Seven trials comprising heterogeneous antibiotic and probiotic therapies showed a general benefit in eradication rates due to probiotic supplementation, but in two only, the results were statistically significant, thus concluding that no convincing evidence was present to support the use of probiotics with triple therapy in children [47]. Moreover, in 2015, the Latin American Expert group consensus stated that at that time, there was a lack of sufficient evidence to recommend the administration of probiotics in this area [48]. However, other evidences suggest that some probiotics like *S. boulardii* or *L. casei* alone or multistrains (*L. acidophilus* + *B. bifidum*, *B. mesentericus* + *C. butyricum* + *S. faecalis*) can be helpful to treat *Hp* and prevent side effects due to the eradication therapy in pediatric patients, recommending their use [49–51].

**Figure 1:**
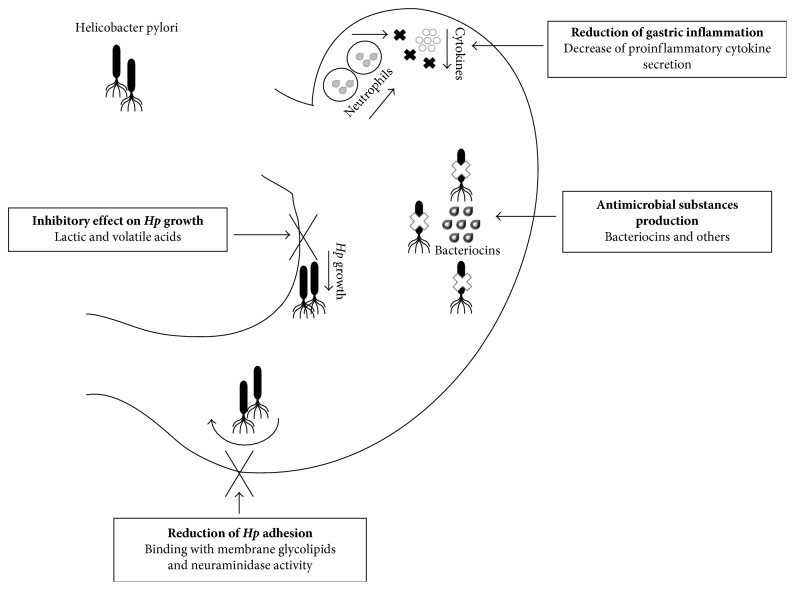
Mechanisms of action of probiotics.

The beneficial effects of these probiotics against *Hp* infection can be related to their intrinsic properties that can depend on probiotic species (Figure 1). *Lactobacilli* have been shown to possess the following properties:

1. Production of antimicrobial substances, as bacteriocins that are synthesized by ribosomes and secreted by several bacteria including *Lactobacilli*. These substances are provided with antimicrobial activity and represent an important and successful weapon against other microbial species like *Hp* [13, 52]. Some bacteriocins, such as nisin, pediocin, acidocin, and lacticin, contribute to homeostasis of the complex GI tract ecosystem [53–55].
2. Capacity to survive in the gastric acidic environment by inducing lactic and volatile acids' production which has an inhibitory effect on *Hp* growth. The effects of supplementation with *L. salivarius* have been documented in a mouse model study, and its efficacy has been related to the high amount of lactic acid produced by the bacterium that interferes with the urease activity of the pathogen [56]. Furthermore, in vitro, *L. salivarius* is able to reduce gastric inflammation by modulating local cytokine secretion, in particular of IL-8 directly related to neutrophil recruitment and mucosal inflammation, probably as a response to the suppressed secretion of *CagA* virulence factor [57].
3. Ability to adhere at gastric and duodenal cells and thus to perform a competitive action against pathogens. Mukai et al. found that two *L. reuteri* strains, *JCM 1081* and *TM105*, were able to bind specific membrane glycolipids and therefore occupy the potential site of *Hp* adhesion, inhibiting this pathogen gastric colonization [58].

Similar mechanisms of action were also described for other probiotics. *Bifidobacterium* strains are able to produce antimicrobial substances that can inhibit *Hp* growth, thus improving eradication rates [10, 44, 59]. Interestingly, a study in mice by Yu et al. suggested that a probiotic mixture containing *B. longum*, *L. acidophilus*, and *E. faecalis* can ameliorate *Hp* gastritis reducing the inflammatory response by inhibiting cytokine secretion such as IL-8, TNF-*α*, G-CSF, and GM-CSF [60]. Conversely, *S. boulardii* can reduce *Hp* adhesion at gastric and duodenal cells through its neuraminidase activity able to selectively reduce the expression of *α* (2-3)-linked sialic acid on cell surface, a target of *Hp* adhesions [61].

## 4. Conclusion

Our review highlights how some probiotic strains can improve *Hp* eradication rates and prevent antimicrobial therapy side effects, likely due to the capabilities of these microorganisms to directly act against *Hp*, restoring a healthy microbiota. In Italy, the mean cost of probiotics in addition to antimicrobial therapy based on the studies present in this review is about 2 € per day of treatment per patient. In our opinion, this cost is widely justified by the reduction of side effects due to antibiotic therapy and the increased eradication rate which reduces the need for a second-line treatment. Therefore, the administration of probiotics may be considered an adjunctive treatment especially when *Hp* eradication fails. However, not all probiotics are alike useful, and probably, the curative effects are strain dependent. Additional studies are necessary to better understand their role in *Hp* infection, particularly when using the updated eradication schedules.

At present, few studies or meta-analysis are available on a direct comparison between beneficial effects of different probiotics species during antimicrobial therapy. However, according to the available data and to the Maastricht V Consensus Report statement “Certain probiotics may have a beneficial effect on *Hp* eradication” [30], we recommend the use of *Lactobacillus*, *Bifidobacterium*, and *Saccharomyces* strains. Nevertheless, in our opinion, more trials with probiotics should be tailored to the state of gastric microbiota composition before and after *Hp* eradication therapy, inasmuch as it could explain the role of various bacteria in the development of gastric diseases.

## Figures and Tables

**Table 1:** Efficacy of probiotics administration in addiction to antimicrobial therapy compared to antimicrobial therapy alone in clinical trials present in our review.

Study	Patients	Antimicrobial therapy	Probiotic	Duration	Eradication improvement	Side-effects reduction
Ojetti et al. [33]	Adults	E: 20 mg bid,	*Lactobacillus reuteri* (1 × 10^8^ CFU tid)	14 days	Yes	Yes
		L: 500 mg bid,				
		A: 1 g bid				
Armuzzi et al. [34]	Adults	P: 40 mg bid,	*Lactobacillus* GG (6 × 10^9^ CFU bid)	14 days	No	Yes
		C: 500 mg bid,				
		T: 500 mg bid				
Chitapanarux et al. [39]	Adults	E: 40 mg bid,	*Bifidobacterium longum* (not specified)	4 weeks	Yes	Yes
		C: 500 mg bid,				
		A: 1 g bid				
Yasar et al. [40]	Adults	P: 40 mg bid,	*Bifidobacterium DN-173* 010-1010 CFU/g yogurt 125 ml	14 days	No	Yes
		C: 500 mg bid,				
		A: 1 g bid				
Song et al. [41]	Adults	O: 20 mg bid,	*Saccharomyces boulardii* (3 × l0^10^ CFU/g)	4 weeks	Yes	Yes
		C: 500 mg bid,				
		A: 1 g bid				
Du et al. [43]	Adults	O: 20 mg bid,	*Lactobacillus acidophilus* (5 × 10^6^), *Streptococcus faecalis* (2.5 × 10^6^), and *Bacillus subtilis* (5 × 10^3^) tid	2 weeks	Yes	No
		C: 500 mg bid,				
		A: 1 g bid				

O: omeprazole; P: pantoprazole; E: esomeprazole; C: clarithromycin; A: amoxicillin; L: levofloxacin; T: tinidazole.
